# Efficacy of Adding Clonidine to Bupivacaine 0.25% Versus Plain Bupivacaine 0.25% Infiltration in Scalp Blocks for Supratentorial Craniotomy

**DOI:** 10.7759/cureus.85274

**Published:** 2025-06-03

**Authors:** Chris Leslie Lemos, Anivesh Jain, Chhavi Dwivedi, Pradyumna Singh Kakodia, Aparna Tamaskar

**Affiliations:** 1 Department of Neuroanaesthesia, Superspeciality Hospital, Netaji Subhash Chandra Bose Medical College, Jabalpur, IND; 2 Department of Anaesthesiology, Superspeciality Hospital, Netaji Subhash Chandra Bose Medical College, Jabalpur, IND

**Keywords:** analgesia, bupivacaine, clonidine, scalp blocks, supratentorial craniotomy

## Abstract

Background

Scalp blocks help attenuate hemodynamic responses and provide analgesia in neurosurgical procedures. Clonidine, an alpha-2 agonist, has been known to enhance the duration of analgesia when used as an adjuvant in peripheral nerve blocks. Our aim was to evaluate the efficacy of adding clonidine 2 mcg/kg to bupivacaine 0.25% versus plain bupivacaine 0.25% in scalp block infiltration in patients undergoing supratentorial craniotomy by comparing the duration of analgesia and perioperative hemodynamic parameters in both groups.

Methods

We enrolled 60 patients in this study based on our inclusion criteria. Patients were divided equally into two groups. Scalp blocks were administered after induction of general anaesthesia. Group A comprised 30 patients and received plain 0.25% bupivacaine infiltration. Group B consisted of 30 patients and received clonidine 2 mcg/kg with 0.25% bupivacaine scalp block infiltration. Heart rate (HR) and mean arterial pressure (MAP) were recorded from application of Mayfield® pins (Integra Lifesciences, Princeton, NJ) every five minutes till the opening of the dura and every five minutes from dura closure up to completion of procedures. Postoperatively, heart rate and MAP were recorded hourly up to four hours. Pain was assessed in terms of a numeric rating scale (NRS) every four hours for the first 24 hours (score 0: no pain; score 10: unbearable pain). A score of 3 was considered a threshold for administration of rescue analgesia. Intraperative hemodynamic complications were noted.

Results

Group B has significantly lower NRS scores at 4 and 8 hours postoperatively. Group B also had significantly longer time before rescue analgesia at 792 +/- 190.8 minutes (13.2 +/- 3.18 hours) compared to Group A at 415.8 +/- 155.4 minutes (6.93 +/- 2.59 hours, p< 0.001). MAP was significantly lower in group B during the first 30 minutes from the pin application, however, there was no statistical difference in the heart rate between both the groups during this time interval. We observed a significantly lower heart rate in group B from 15 minutes onwards after dura closure, and this continued in the postoperative period. Mean arterial pressure was comparable between the groups until closure. Group B demonstrated a significantly lower MAP from the second hour in the postoperative period. Overall paracetamol consumption in the postoperative period and fentanyl requirement intraoperatively were also significantly higher in group A.

Conclusion

Addition of clonidine to bupivacaine in scalp blocks significantly (p<0.001) enhanced analgesic efficacy and hemodynamic control in patients undergoing supratentorial craniotomy procedures.

## Introduction

Anesthesia for neurosurgical procedures is tailored to achieve a balance between intracranial tension, cerebral metabolic rate, and cerebral blood flow [1]. Mayfield® pins (Integra Lifesciences, Princeton, NJ) application causes intense pain and leads to an abrupt rise in heart rate and arterial blood pressure [2]. General anesthesia, when supplemented with scalp blocks, helps attenuate response to pins and incision, leading to stable hemodynamics and providing analgesia perioperatively and extending postoperative benefits like early mobilisation and early discharge [3,4]. Multiple adjuvants have been used in peripheral nerve blocks to enhance their effects and duration of action. Clonidine has been used through multiple routes, such as intravenous, intrathecal, epidural, and as an adjuvant with local anesthetics. It is an alpha-2 agonist which causes hypotension, provides extended duration of analgesia, and reduces stress response to surgery [5]. Efficacy of the addition of clonidine to scalp blocks has been reviewed previously by Wajekar et al. [3]. Due to the scarcity of data in the Indian population, we aimed to study the effects of adding clonidine 2 mcg/kg body weight to 0.25% bupivacaine infiltration in scalp blocks in supratentorial craniotomy surgery to assess its effects on perioperative hemodynamic responses and duration of analgesia to help devise institutional protocol in these surgical procedures.

## Materials and methods

This prospective, single-centre, double-blinded, randomised comparative study was conducted at our institute after receiving approval from the Institutional Ethics Committee at Netaji Subhash Chandra Bose Medical College, Jabalpur, India (No. IEC/2022/4406) and registration with the Clinical Trials Registry of India (CTRI/2022/11/047065). Our primary objective was to evaluate the efficacy of the addition of clonidine 2 mcg/kg to scalp blocks with bupivacaine 0.25% on duration of postoperative analgesia, while the effects on intraoperative hemodynamic parameters like heart rate and mean arterial blood pressure and anaesthesia requirements in patients undergoing supratentorial craniotomy surgery were the secondary objectives.

We selected a clonidine dose of 2 mcg/kg body weight based on a prior study by Wajekar et al., where the mean time to first rescue analgesia was reported as 408.17 ± 209.81 minutes in the control group and 887.97 ± 398.21 minutes in the clonidine group, indicating a substantial difference in postoperative analgesia duration [3]. Using these values, we calculated the required sample size, setting the confidence level at 95% and the study power at 95% using OpenEpi version 3 software (Open Source Epidemiologic Statistics for Public Health). The resulting calculation suggested that 12 participants per group (total n=24) would be sufficient to detect a statistically significant difference. However, to enhance the precision of our findings and improve the reliability of the results, we increased the sample size to 60 participants, with 30 individuals allocated to each group. 

In this study, we recruited patients aged 18 to 60 years, American Society of Anesthesiologists (ASA) Class I & II, and with a preoperative Glasgow Coma Scale of 15, posted for supratentorial craniotomy procedure after written and informed consent. We excluded patients with a history of hypertension, diabetes mellitus, ischemic heart disease, bradyarrhythmias, allergy to any of the study drugs, emergency surgery, previous history of craniotomy, and patients refusing to participate in the study. After a comprehensive pre-anesthetic check, patients were randomly divided into two groups of 30 each. Group A received 20 ml of 0.25% bupivacaine, and Group B received 20 ml of 0.25% bupivacaine with the addition of clonidine 2 mcg/kg in scalp blocks. To achieve blinding, an equivalent amount of saline was added to bupivacaine in Group A. The bupivacaine dose was restricted to <2 mg/kg. Double blinding was achieved by using pre-coded medications. Participants and the anesthetist involved in intraoperative care and postoperative data collection were blinded to the groups. After establishing monitoring with a five-lead electrocardiogram, noninvasive blood pressure, end tidal carbon dioxide, temperature, and pulse oximetry, anesthesia was induced with midazolam 0.03 mg/kg, fentanyl 2 mcg/kg, propofol 2 mg/kg, and vecuronium 0.1 mg/kg. An appropriately sized endotracheal tube was secured and anesthesia maintenance was achieved by an air and oxygen mixture (70:30), isoflurane 0.7 to 1.2 minimal alveolar concentration (MAC) range with vecuronium infusion (0.01 to 0.015 mg/kg/hr). Bilateral scalp blocks were administered using 25 gauge needle with the drugs specific to the assigned groups to block supraorbital and supratrochlear nerves near the supraorbital groove, zygomaticotemporal nerve near the lateral canthus of the eye, auriculotemporal nerve near the tragus and infiltrating along the line connecting mastoid process with the occipital protuberance to block lesser and greater occipital nerves posteriorly. Incision site infiltration with lignocaine 2% with adrenaline (1:200000) was administered by the surgeon, limiting its dose to <5 mg/kg.

Intraoperative hemodynamics, including heart rate (HR) and mean arterial pressure (MAP), were recorded every five minutes starting with the application of Mayfield® pins, and after dura closure, up to completion of the procedure. Additionally, intraoperative fentanyl use and complications (bradycardia, tachycardia, hypertension, hypotension, blood loss) were noted. Deviation of heart rate and mean arterial pressure > 20% from baseline was treated. At the end of the procedure, patients were extubated after fulfilling the extubation criteria. Patients with surgical complications, requiring postoperative ventilatory support, and with new-onset neurodeficits were withdrawn from the study. Postoperatively, heart rate and mean arterial pressure were recorded hourly for four hours. Pain was assessed using a numeric rating score (NRS) (0: no pain, -10: worst possible pain), with a score of 3 being the threshold to administer rescue analgesic paracetamol 1 gm, and time was noted. Intraoperative reduction in MAP (<20%) was treated with ephedrine 6 mg boluses and bradycardia (HR < 50/min) with glycopyrrolate 0.004 mg/kg. Increase in MAP (>20%) was treated with 0.5 mcg/kg fentanyl and further management with labetalol boluses. Patients with intraoperative complications like bleeding and hemodynamic instability requiring inotropic or vasopressor support, and with surgical complications, were discontinued from the study.

Statistical analysis

All procedures were completed within the planned timeframe. The data was collected systematically, compiled, and organized into tables. Statistical analysis was performed using GraphPad Prism version 10.4.2 (633) for Windows (GraphPad software, Boston, Massachusetts, USA, www.graphpad.com). Quantitative variables were compared using the unpaired Student’s t-test and presented as mean ± standard deviation (SD). Categorical variables were analyzed using the Chi-square test. Normality of the data was assessed with the Kolmogorov-Smirnov test. A p-value of less than 0.05 was considered statistically significant.

## Results

This study included 60 patients who were scheduled for elective supratentorial craniotomy surgery. They were divided into two groups of 30 each, Group A receiving bupivacaine 0.25% and Group B receiving bupivacaine 0.25% with clonidine 2 mcg/kg in scalp blocks. Both groups were comparable in terms of demographic data like age, sex, weight, height, and ASA class of patients, with no statistical difference as shown in Table 1.

**Table 1:** Demographics *Statistically significant if p<0.05 ASA: American Society of Anesthesiologists, t: unpaired t test, X^2^: Chi-square test.

Parameter	Group A (n=30) Mean +/-SD	Group B (n=30) Mean +/- SD	Statistical test	p-Value	Significance
Age (years)	35.23 +/- 12.06	35.47 +/- 12.04	t = 0.074	0.94	No
Weight (kg)	62.4 +/- 9.08	62.16 +/- 8.91	t = 0.101	0.92	No
Height (cm)	164.46 +/- 7.90	164.76 +/- 7.68	t = 0.149	0.882	No
Sex (male/female)	18/12	16/14	X^2^ = 0.271	0.602	No
ASA physical status (I/II)	19/11	17/13	X^2 ^= 0.278	0.598	No
Duration of surgery (min)	158 +/- 24.79	161.83 +/- 25.67	t = 0.582	0.559	No

Comparison of hemodynamic parameters between both groups is shown in Table 2. Baseline heart rate and mean arterial pressure values were comparable in both groups. MAP was significantly lower in group B during the first 30 minutes after pin application. However, there was no statistical difference in the heart rate between the groups during this time interval. We observed a significantly lower heart rate in group B from 15 minutes onwards after dura closure, and this continued in the postoperative period. Mean arterial pressure was comparable between the groups until closure.

**Table 2:** Heart rate and mean arterial pressure in both groups P0: time of Mayfield® pins applications, D0: time of dura closure, *statistically significant if p<0.05, t: unpaired t test, X^2^: Chi-square test, HR: Heart rate, MAP: Mean arterial pressure. Mayfield® pins (Integra Lifesciences, Princeton, NJ).

	Group A HR Mean +/- SD	Group B HR Mean +/- SD	Statistical Test	P Value	Group A MAP Mean +/- SD	Group B MAP Mean +/- SD	Statistical Test	P Value
Baseline	79.06 +/- 5.92	78.7 +/- 6.87	t = 0.231	0.8183	87.93 +/- 4.32	88.1 +- 4.52	t = 0.146	0.8845
P0	80.86 +/- 10.47	80.2 +/- 10.12	t = 0.251	0.8030	88.3 +/- 912	83.866 +/- 787	t = 2.015	0.0486*
P5	80.9 +/- 11.786	80.2 +/- 10.924	t = 0.239	0.8123	87.93 +/- 8.21	83.33 +/- 7.077	t = 2.323	0.0238*
P10	79.66 +/- 10.25	79.9 +/- 12.652	t = 0.078	0.9377	86.9 +/- 8.809	82.4 +/- 6.088	t = 2.302	0.0254*
P15	79.26 +/- 11.70	79.76 +/- 10.81	t = 0.172	0.8641	85.57 +/- 8.152	81.07 +/- 6.554	t = 2.356	0.0220*
P20	79.23 +/- 11.27	79.86 +/- 11.43	t = 0.216	0.8298	84.77 +/- 7.80	80.53 +/- 6.08	t = 2.344	0.0227*
P25	79.4 +/- 10.01	79 +/- 10.74	t = 0.149	0.8819	84.07 +/- 7.07	80.13 +/- 6.846	t = 2.189	0.0327*
P30	79 +/- 9.23	78.53 +/- 9.008	t = 0.198	0.8436	83.77 +/- 7.37	80.23 +/- 6.021	t = 2.033	0.0468*
P35	79.1 +/- 9.35	77.66 +/- 8.33	t = 0.627	0.5334	83.40 +/- 7.22	80.10 +/- 6.59	t = 1.849	0.0696
P40	79.46 +/- 9.30	77.4 +/- 7.28	t = 0.959	0.3418	82.73 +/- 7.10	79.933 +/- 5.77	t = 1.691	0.0964
D0	81.53 +/- 11.13	77.57 +/- 8.48	t = 1.552	0.1264	83.13 +/- 9.01	81.77 +/- 7.31	t = 0.645	0.5216
D5	81.47 +/- 9.69	78.37 +/- 7.87	t = 1.360	0.1793	82.6 +/- 8.40	80.63 +/- 6.95	t = 0.988	0.3273
D10	81.36 +/- 9.73	78.20 +/- 7.02	t = 1.445	0.1544	82.83 +/- 8.07	80.43 +/- 6.66	t = 1.257	0.2141
D15	82.03 +/- 9.11	77.63 +/- 7.29	t = 2.066	0.0435*	82.77 +/- 7.49	79.33 +/- 6.67	t = 1.874	0.0660
D20	81.70 +/- 8.404	77.6 +/- 7.29	t = 2.301	0.0259*	82.33 +/- 7.38	78.73 +/- 6.94	t = 1.944	0.0567
D25	81.37 +/- 8.30	76.67 +/- 7.25	t = 2.335	0.0231*	82.07 +/- 7.33	78.80 +/- 6.61	t = 1.812	0.0754
D30	81.20 +/- 7.19	76.7 +/- 7.12	t = 2.435	0.0180*	82.2 +/- 7.43	78.60 +/- 6.82	t = 1.911	0.0611
D35	80.83 +/- 7.49	76 +/- 7.54	t = 2.489	0.0157*	81.67 +/- 7.43	78.13 +/- 7.30	t = 1.857	0.0683
D40	80.07 +/- 7.78	75.33 +/- 8.21	t = 2.291	0.0256*	81.13 +/- 7.011	77.87 +/- 7.47	t = 1.746	0.0861
Post op 1hr	81.83 +/- 8.89	77.13 +/- 8.35	t = 2.110	0.0392*	83.5 +/- 6.600	80.43 +/- 6.317	t = 1.838	0.0711
Post op 2hr	80.13 +/- 6.38	73.4 +/- 6.866	t = 3.934	0.002*	83.66 +/- 7.048	79.96 +/- 5.22	t = 2.309	0.0248*
Post op 3hr	79.13 +/- 8.33	72.866 +/- 7.51	t = 3.058	0.0034*	84.2 +/- 7.312	80.4 +/- 4.76	t = 2.384	0.0210*
Post op 4hr	78.33 +/- 7.33	73.4 +/- 7.57	t = 2.770	0.0075*	82.56 +/- 7.21	79.033 +/- 5.79	t = 2.090	0.0412*

**Figure 1:**
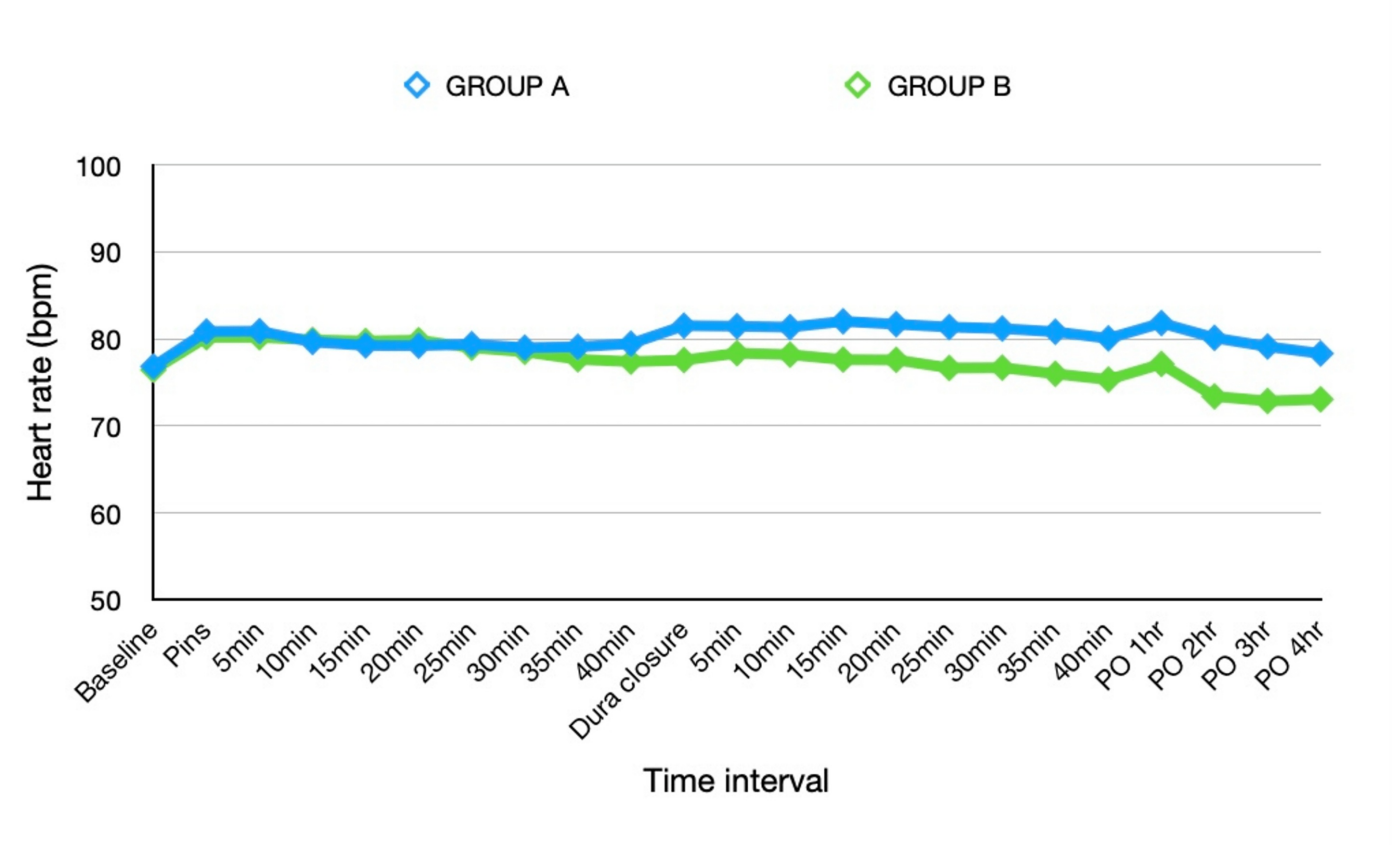
Comparison of Heart rate (beats per minute) in both groups

**Figure 2:**
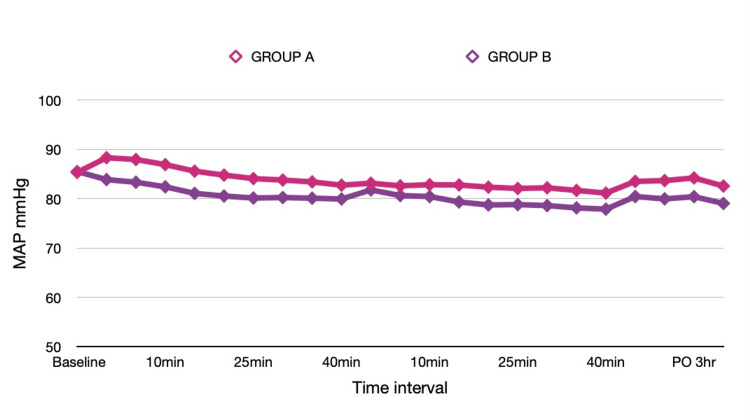
Comparison of mean arterial pressure (mmHg) in both groups

**Figure 3:**
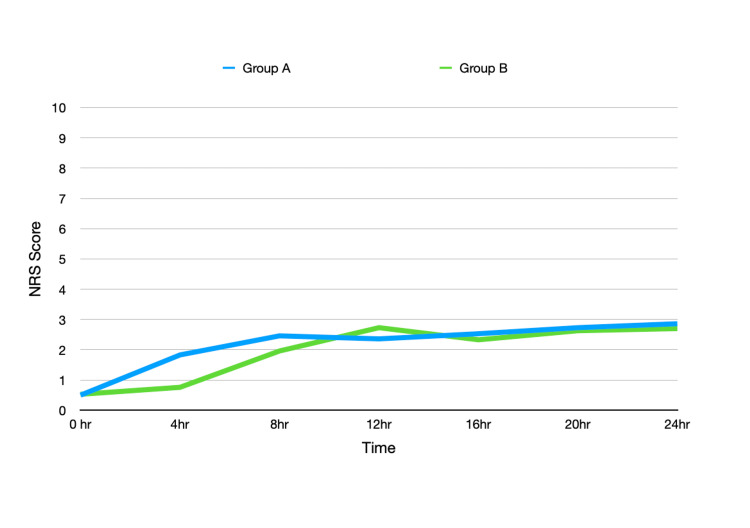
Postoperative numeric rating score (NRS) in both groups

Group B has significantly lower NRS scores at four and eight hours postoperatively. Comparison of NRS scores between both groups at different points is depicted in Figure 3.

**Figure 4:**
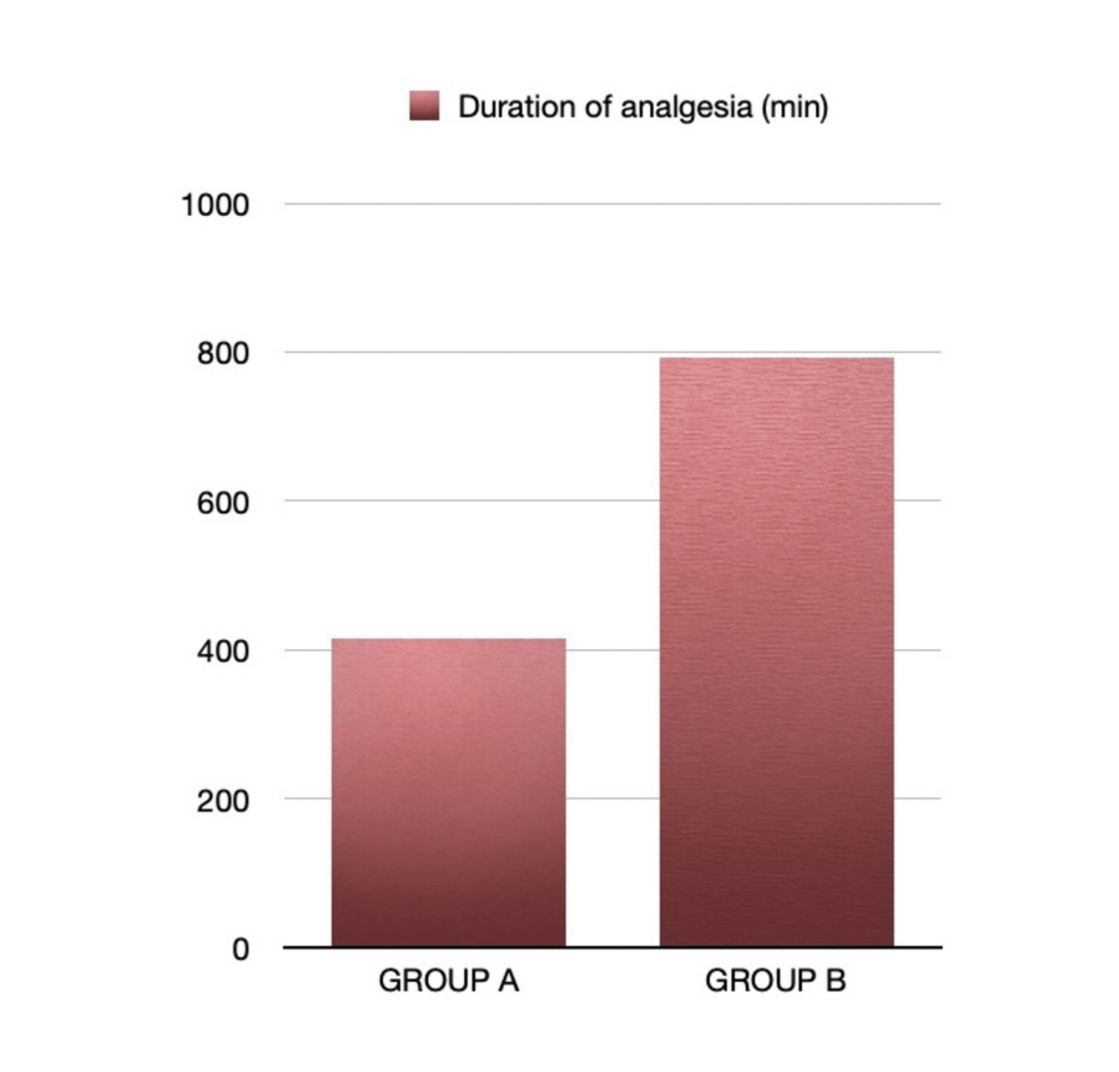
Time to rescue analgesia in both groups (minutes)

Group B also had significantly longer time before rescue analgesia at 792+190.8 minutes (13.2 +3.18 hours) 95% CI [12.01, 14.39] compared to Group A at 415.8 +155.4 minutes (6.93 +2.59 hours) 95% CI [4.35, 9.51] (p< 0.001) as shown in Figure 4.

**Figure 5:**
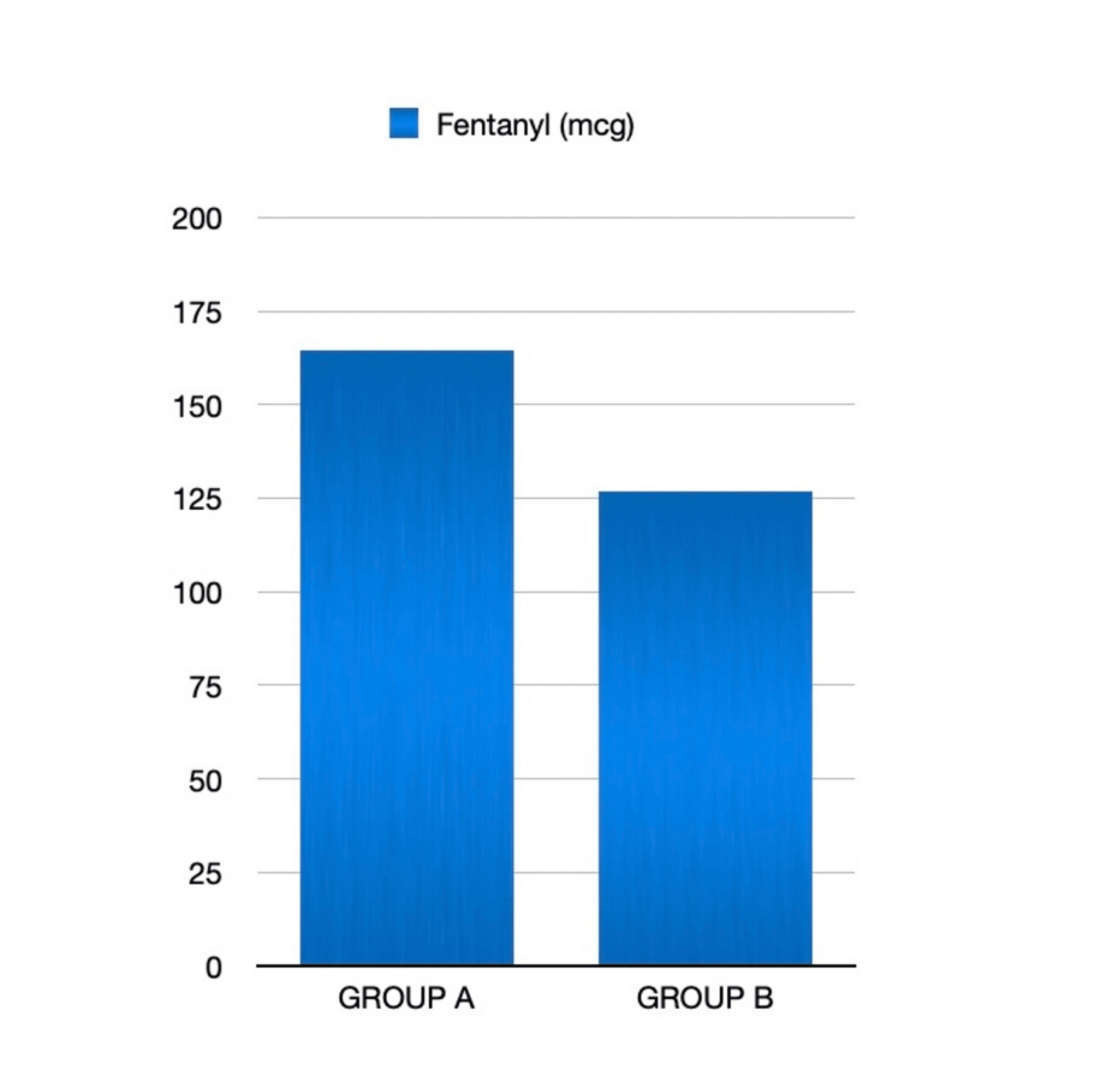
Intraoperative fentanyl requirement in both groups (mcg)

**Figure 6:**
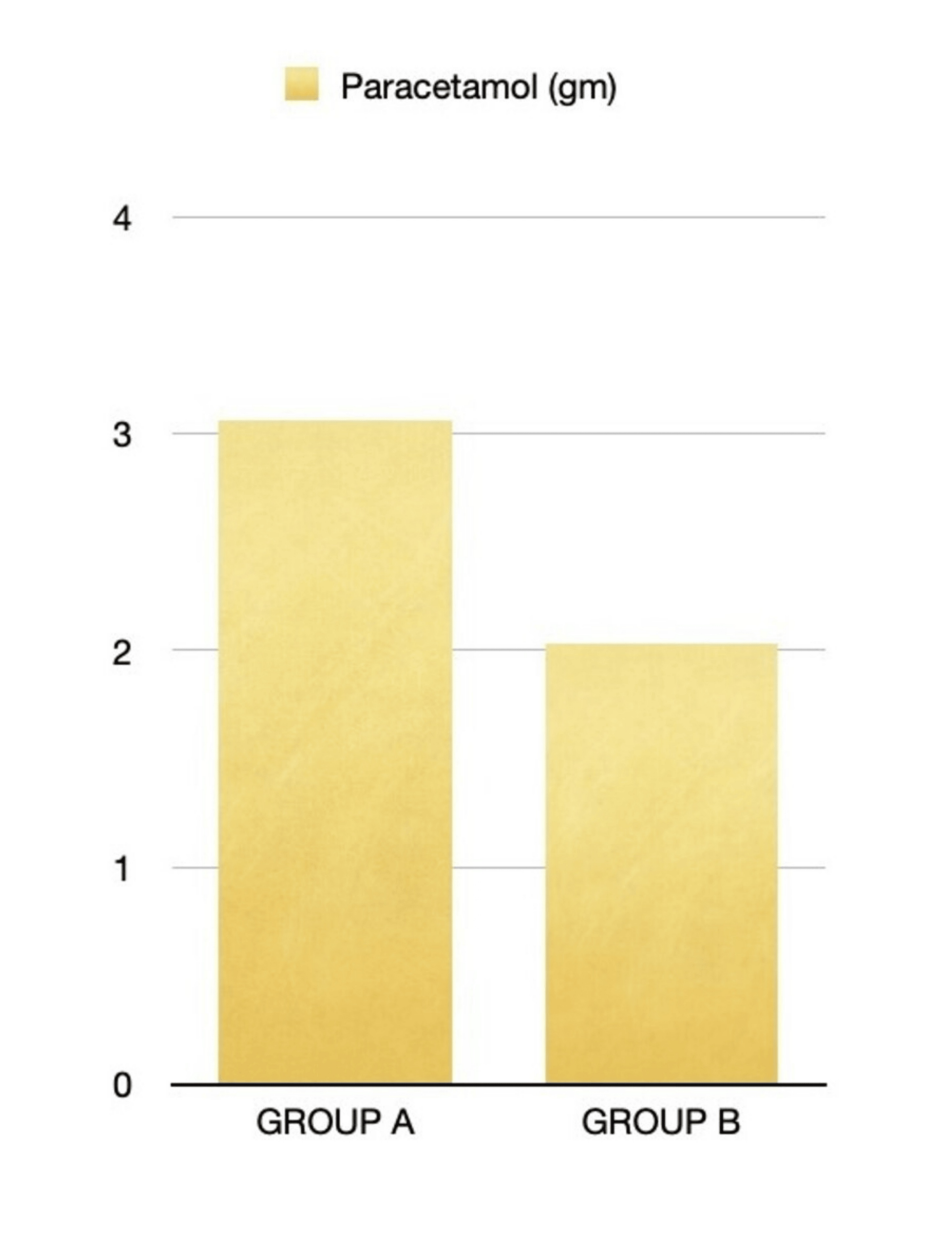
Postoperative paracetamol requirement in both groups (g)

Overall paracetamol consumption in the postoperative period and fentanyl requirement intraoperatively were also significantly higher in Group A, as shown in Figures 5, 6. In group A, 11 patients required fentanyl 0.5 mcg/kg boluses to treat MAP >20% increase from baseline, which led to significantly higher fentanyl use compared to group B. In group B, three patients had hypotension requiring an ephedrine bolus.

## Discussion

Scalp blocks are being increasingly used in neurosurgical procedures to attenuate perioperative hemodynamic responses and provide analgesia [6-8]. They can have an opioid sparing effect, which can be beneficial in faster emergence and recovery in patients undergoing neurosurgical procedures. We observed a significantly prolonged duration of analgesia in Group B, 13.2 +3.18 hours, compared to Group A, which had a time to rescue analgesia of 6.93 + 2.59 hours, attributed to prolongation of block by clonidine. Time to rescue analgesia was noted when the NRS score was 3. However, we continued pain assessment in patients up to 24 hours. In this study, we found a significant reduction in MAP during the first 30 minutes following pin application in group B compared to group A. This difference can be attributed to systemic absorption of clonidine. Postoperative MAP from the second hour onwards was also significantly lower in group B. Heart rate was comparable between the groups during this time duration; however, Group B demonstrated a significant reduction around 15 minutes after dura closure, which continued in the post-operative period. Overall, fentanyl consumption was also significantly lower in group B due to lower MAP levels. 

The role of scalp blocks has also been evaluated in the management of stress response to surgery [9]. Carella et al. found significantly lower hemodynamic parameters during noxious stimuli and prolonged duration of postoperative analgesia in the group of patients who received scalp blocks with levobupivacaine undergoing craniotomy [10]. Clonidine is an alpha-2 agonist that reduces heart rate and blood pressure. It exerts effects centrally and peripherally. It has been proven to improve the efficacy of peripheral nerve blocks as an adjuvant in terms of analgesia duration by intrinsic blockade of A delta and C fibres [11]. Addition of clonidine to scalp blocks leads to slow systemic release, which helps in managing the pressor responses to surgical stimuli like the Mayfield® pins application in surgery [3]. 

Numerous studies have studied the effect of oral clonidine on hemodynamic response to pin insertion and incision in patients undergoing craniotomy [12,13]. Costello and Cormack reported effective obtundation of pressor responses was achieved in the oral clonidine 3 mcg/kg group compared with placebo [12]. Clonidine also slows down vascular uptake of bupivacaine by inducing local vasoconstriction thus prolonging duration of analgesia.

Wajekar et al. studied the effects of clonidine 2 mcg/kg as an adjuvant to scalp blocks with 0.25 % bupivacaine and found significantly prolonged duration of analgesia (887.97 + 398.21 mins) compared to infiltration with plain 0.25% bupivacaine (408.17 + 209.81 mins) [3]. They also reported better hemodynamic stability with clonidine in the perioperative period. Bagle et al. compared scalp blocks with ropivacaine 0.5% vs clonidine 1 mcg/kg with ropivacaine 0.5% and found that duration of analgesia was significantly prolonged in the clonidine + ropivacaine group (9.10 + 1.4 hrs) as compared to the ropivacaine group (4.30 + 1.5 hrs) [14]. In a study reported by Maharani et al., they compared the effects of scalp block with bupivacaine 0.25% plus clonidine 2 mcg/kg (group I) with bupivacaine 0.25% and dexamethasone 8 mg (group II) on postoperative NRS score and cortisol levels. Group I compared to group II had significantly lower NRS score at 12 (2.15+1.13 vs 3.30+1.08) and 24 hours (postoperatively 2.10+1.02 vs 3.45+1.57) [9]. They also found significantly lower cortisol levels in the clonidine group.

Dash et al. in their study compared clonidine 2 mcg/kg as adjuvant with 0.5% bupivacaine in scalp block (Group B) and clonidine 2 mcg/kg IV in addition to scalp block (Group C) and found that in group B, there was a progressive decrease in HR at pin application (8.6% of baseline, p = 0.0063) until 60 minutes, and also from 0 to 35 minutes after dura closure which was statistically significant [15]. There was a significant fall in MAP in both groups during pin application (p value 0.0000 and 0.0001, respectively). The MAP remained below baseline after dural closure, with significant reduction for 30 and 15 minutes after dural closure in groups B and C, respectively. 

Other studies using dexmedetomidine as an adjuvant in scalp blocks also gave similar results [4,16]. Vallapu et al. found significantly longer duration of analgesia in group BDNB, 12 hours (bupivacaine plus dexmedetomidine scalp blocks) versus group BDI, eight hours (bupivacaine with dexmedetomidine wound infiltration) versus group BI, four hours (bupivacaine skin infiltration) in patients undergoing elective craniotomy [16].

Our study has several limitations. This is a single-centre study with a relatively small sample size. We also used a fixed dose of clonidine. Future research can be directed towards the inclusion of the paediatric population and comparing multiple doses of clonidine. Use of larger sample size can also ensure generalizability of results.

## Conclusions

Addition of clonidine to bupivacaine in scalp blocks significantly (p<0.001) enhanced analgesic efficacy and hemodynamic control in patients undergoing supratentorial craniotomy procedures. Hemodynamic control continued in the postoperative period as well. Clonidine also reduces intraoperative consumption of fentanyl owing to stable hemodynamics. This makes clonidine a useful option for use in neurosurgical procedures, which can facilitate faster emergence due to its opioid sparing effects and recovery. These findings are consistent with existing literature and support the clinical utility of clonidine as an adjuvant in scalp blocks for better perioperative analgesia and reduction in opioid use.
